# Low Levels of Neutralizing Antibodies to Influenza A (H5N1) and D Viruses Among Cattle and Cattle Workers on US Farms, 2024–2025

**DOI:** 10.1111/irv.70162

**Published:** 2025-09-08

**Authors:** Ismaila Shittu, Daniel B. Cummings, John T. Groves, Alex G. Hagan, Gregory C. Gray

**Affiliations:** ^1^ Department of Medicine, Division of Infectious Diseases University of Texas Medical Branch Galveston Texas USA; ^2^ Heritage Veterinary Partners Madisonville Tennessee USA; ^3^ Livestock Veterinary Service Eldon Missouri USA; ^4^ Ironsides Animal Health Shelbyville Kentucky USA; ^5^ Institute for Human Infections and Immunity University of Texas Medical Branch Galveston Texas USA; ^6^ Department of Microbiology and Immunology University of Texas Medical Branch Galveston Texas USA

**Keywords:** cattle, cattle workers, influenza a virus, influenza D virus, neutralizing antibodies, one health

Dear Editor,

1

Bovine respiratory disease complex (BRDC) is a major disease problem in cattle production systems. Numerous pathogens have been implicated as causing BRDC, including the recently discovered influenza D virus (IDV) [1]. IDV has been found to be highly enzootic in cattle across multiple continents [1, 2], and there is some evidence of spillover to livestock workers [3, 4]. In March 2024, unprecedented outbreaks of influenza A virus (IAV) H5N1 clade 2.3.4.4b were reported in the United States [5] with occasional spillover to dairy and poultry workers [6, 7]. Given the occupational threats of both IDV and IAV H5N1, we sought to assess the dynamics of antibodies to IAV H5N1 and IDV in farm workers and cattle.

As part of our One Health surveillance initiative in the United States and Mexico [6, 8], between April 2024 and May 2025 we enrolled a total of seven dairy and beef cattle farms in Indiana (*n* = 1), Kentucky (*n* = 3), and Texas (*n* = 2) (Table 1). Our visits to these farms took place on four separate occasions, permitting us to prospectively follow the farms. We obtained informed consent from every farm worker who participated in the study. Based on availability, we collected 5 to 10 mL of blood from both the cattle and the farm workers through venipuncture using a plain vacutainer tube (BD, Franklin Lakes, NJ, USA). After collection, the blood samples were centrifuged at 600 *g* for 15 min, and the serum was harvested and stored in microtubes at −20°C. Throughout the four visits, we collected 270 serum samples, which comprised 142 samples from cattle and 128 samples from farm workers (Table 1). Ethical approval for the study was granted by the University of Texas Medical Branch's Institutional Review Board (23‐0085).

**TABLE 1:** Microneutralization assay results by state, cattle farm type, visit number (one through four) and virus type, during the period April 2024 to May 2025.

State where farms were studied (number of farms)	Cattle farm type	First visit (April – May 2024)			Second visit (July 2024)			Third visit (December 2024)			Fourth visit (May 2025)			Total collected
		Total	IAV (+)	IDV (+)	Total	IAV (+)	IDV (+)	Total	IAV (+)	IDV (+)	Total	IAV (+)	IDV (+)	6
Cattle serum samples														
Indiana (*n* = 1)	Beef	0	0 (0.0%)	0 (0.0%)	0	0 (0.0%)	0 (0.0%)	6	0 (0.0%)	2 (33.3%)	0	0 (0.0%)	0 (0.0%)	39
Kentucky (*n* = 3)	Beef	0	0 (0.0%)	0 (0.0%)	20	0 (0.0%)	0 (0.0%)	19	0 (0.0%)	1 (5.3%)	0	0 (0.0%)	0 (0.0%)	30
Texas (*n* = 1)	Beef	0	0 (0.0%)	0 (0.0%)	0	0 (0.0%)	0 (0.0%)	30	0 (0.0%)	10 (22.2%)	0	0 (0.0%)	0 (0.0%)	67
Texas (*n* = 1)	Dairy	0	0 (0.0%)	0 (0.0%)	22^a^	19 (86.4%)	0 (0.0%)	15^a^	10 (73.3%)	0 (0.0%)	30^a^	7 (23.3%)	1 (3.3%)	142
Total		0	0 (0.0%)	0 (0.0%)	42	19 (45.2%)	0 (0.0%)	70	10 (73.3%)	13 (18.6%)	30	7 (23.3%)	1 (3.3%)	
Cattle worker serum samples														
Indiana	9	0 (0.0%)	1 (11.1%)^b^		9	0 (0.0%)	1 (11.1%)^b^	9	0 (0.0%)	1 (11.1%)^b^	10	0 (0.0%)	1 (10.0%)^b^	37
Kentucky	9	0 (0.0%)	0 (0.0%)		6	0 (0.0%)	0 (0.0%)	6	0 (0.0%)	0 (0.0%)	5	0 (0.0%)	0 (0.0%)	26
Texas	14	2 (14.3%)^c^	0 (0.0%)		18	0 (0.0%)	0 (0.0%)	7	0 (0.0%)	0 (0.0%)	26	0 (0.0%)	0 (0.0%)	65
Total	32	2 (6.3%)^c^	1 (3.1%)		33	0 (0.0%)	1 (3.0%)	22	0 (0.0%)	1 (4.5%)	41	0 (0.0%)	1 (2.4%)	128

*Note:* Unless otherwise indicated, we did not sample the same cows during each visit.

^a^ The same 15 dairy cows were sampled on the second and third visit. On the fourth visit, five had been sold, and only 10 of the original 15 cows were sampled. However, 20 new cows were sampled.

^b^ The same cattle worker on the Indiana beef cattle farm had neutralizing antibodies against IDV during each of the four farm visits. The titers were consistent at 1:40 throughout the study period.

^c^ Of the two cattle workers who tested positive at the first visit, we were unable to retest one at a subsequent visit.

To determine the presence of neutralizing antibodies (NAbs) against IAV‐H5 and IDV in both the cattle and farm workers sera, we conducted microneutralization (MN) assays. Briefly, serum samples were treated overnight (18–20 h) with Receptor Destroying Enzyme II (RDE; Denka Seiken, Tokyo, Japan) according to the manufacturer's instructions. This treatment was necessary to eliminate nonspecific inhibitors that could interfere with the assay. Starting with a dilution of 1:20 to 1:2560 RDE‐treated sera, we used a recombinant H5N1 virus (rg‐A/bald eagle/Florida/W22‐134‐OP/2022 of clade 2.3.4.4b) and a bovine‐origin IDV strain (D/Bovine/Kansas/1‐35/2010) on a monolayer of Madin‐Darby canine kidney (MDCK; ATCC cat. no. CRL‐CCL34) cells in 96‐well plates following standard procedures with minor modifications [4, 9].

As measured by the MN assay, none of the enrolled beef cattle farm workers had NAbs antibodies to IAV‐H5 during any of the four visits to the farms in Indiana, Kentucky, and Texas, except for the first visit to Texas (Table 1). During this visit, two dairy farm workers were found to have NAbs to IAV‐H5 [6]. Furthermore, NAbs to IAV‐H5 were not detected in any beef cattle tested during the four field visits in Indiana, Kentucky, and Texas. However, 19 of the 22 (73.3%) Texas dairy cattle on one farm showed elevated NAbs to IAV‐H5 (Table 1). Additionally, at least 7 of 15 (46.7%) out of these dairy cattle maintained elevated NAbs levels during subsequent visits for over a year (Shittu et al., manuscript under journal review).

We found NAbs to IDV in one of 128 farm worker serum samples (0.8%). Notably, antibodies to IDV were consistently detected in the serum of this worker at every visit. This indicates that NAbs in individuals infected with IDV can persist for more than a year. To the best of our knowledge, this is the first report documenting the persistence of antibodies to IDV in farm workers. This finding highlights the importance of monitoring immune responses in this population, who are often exposed to various pathogens in their work environment.

Understanding how long antibodies remain detectable can provide valuable insights into the immune system's effectiveness to clear invading pathogens and guide future health strategies for workers in agricultural settings. In the cattle, during the winter of 2024 (third visit), we detected NAbs against IDV exclusively in the beef cattle (Table 1). In Indiana, 2 of 6 beef cows (33.3%) tested positive for IDV NAbs. In Kentucky, 1 of 19 beef cows (5.3%) had IDV NAbs, while in Texas, 10 of 30 beef cows (22.2%) were found to have IDV NAbs (Table 1). Unlike IAV and other respiratory viruses with high incidences during winter [10], little is known about the seasonality of IDV. More extensive research should be conducted in establishing the seasonality of IDV in cattle to aid in control strategies. In conclusion, our study documents a low prevalence of neutralizing antibodies to IAV H5N1 and IDV in both cattle and cattle workers.

## Author Contributions

**Ismaila Shittu:** investigation, methodology, validation, writing – original draft, writing – review and editing, data curation, formal analysis, conceptualization, visualization. **Daniel B. Cummings:** investigation, methodology, writing – review and editing. **John T. Groves:** investigation, methodology, writing – review and editing. **Alex G. Hagan:** investigation, methodology, writing – review and editing. **Gregory C. Gray:** writing – original draft, writing – review and editing, conceptualization, funding acquisition, project administration, resources, supervision, methodology, investigation.

## Conflicts of Interest

The authors declare no conflicts of interest.

## Data Availability

The data that support the findings of this study are available from the corresponding author upon request.
